# *Anopheles* Salivary Gland Architecture Shapes *Plasmodium* Sporozoite Availability for Transmission

**DOI:** 10.1128/mBio.01238-19

**Published:** 2019-08-06

**Authors:** Michael B. Wells, Deborah J. Andrew

**Affiliations:** a Department of Cell Biology, Johns Hopkins School of Medicine, Baltimore, Maryland, USA; b Johns Hopkins Malaria Research Institute, Johns Hopkins Bloomberg School of Public Health, Baltimore, Maryland, USA; NIAID/NIH

**Keywords:** malaria, mosquito, salivary gland, sporozoite

## Abstract

*Plasmodium* sporozoites (SPZs) must traverse the mosquito salivary glands (SGs) to reach a new vertebrate host and continue the malaria disease cycle. Although SGs can harbor thousands of sporozoites, only 10 to 100 are deposited into a host during probing. To determine how the SGs might function as a bottleneck in SPZ transmission, we have characterized Anopheles stephensi SGs infected with the rodent malaria parasite Plasmodium berghei using immunofluorescence confocal microscopy. Our analyses corroborate findings from previous electron microscopy studies and provide new insights into the invasion process. We identified sites of SPZ accumulation within SGs across a range of infection intensities. Although SPZs were most often seen in the distal lateral SG lobes, they were also observed in the medial and proximal lateral lobes. Most parasites were associated with either the basement membrane or secretory cavities. SPZs accumulated at physical barriers, including fused salivary ducts and extensions of the chitinous salivary duct wall into the distal lumen. SPZs were observed only rarely within salivary ducts. SPZs appeared to contact each other in many different quantities, not just in the previously described large bundles. Within parasite bundles, all of the SPZs were oriented in the same direction. We found that moderate levels of infection did not necessarily correlate with major SG disruptions or abundant SG cell death. Altogether, our findings suggest that SG architecture largely acts as a barrier to SPZ transmission.

## Abstract (precis)

Malaria continues to have a devastating impact on human health. With growing resistance to insecticides and antimalarial drugs, as well as climate change predictions indicating expansion of vector territories, the impact of malaria is likely to increase. Additional insights regarding pathogen migration through vector mosquitoes are needed to develop novel methods to prevent transmission to new hosts. Pathogens, including the microbes that cause malaria, must invade the salivary glands (SGs) for transmission. Since SG traversal is required for parasite transmission, SGs are ideal targets for transmission-blocking strategies. The work presented here highlights the role that mosquito SG architecture plays in limiting parasite traversal, revealing how the SG transmission bottleneck is imposed. Further, our data provide unprecedented detail about SG-sporozoite interactions and gland-to-gland variation not provided in previous studies.

## INTRODUCTION

Malaria remains the vector-borne disease most harmful to human health, causing more than 400,000 annual deaths from hundreds of millions of annual infections (1). This impact comes despite gains made against the malaria death toll over the past 15 years, resulting primarily from insecticide spraying and bed net treatment campaigns. Unfortunately, these gains have diminished over the past 5 years with the rise of insecticide resistance (2). Research into new insecticides, potential malaria vaccines, and genetic strategies is ongoing and holds great promise (3–5).

The identification of mosquitoes as the vectors for *Plasmodium* (6) marked the beginning of research to understand their complex relationship. Much is now known about the journey of the malaria parasite through the mosquito (7–9). Female mosquitoes acquire *Plasmodium* during a blood meal from a previously infected host. Following gamete differentiation and fertilization, the ookinete traverses the midgut epithelium and forms an oocyst on the outer surface. Thousands of sporozoites (SPZs) develop within the oocyst and are released during oocyst rupture and carried by the hemolymph throughout the body cavity. Sporozoites selectively bind to and invade the salivary glands (SGs), where they accumulate in the secretory cavities, the shared lumen, and the salivary duct. SPZs can then be transmitted to a new host along with saliva during subsequent blood meals (8, 10).

The SGs are a key tissue within the vector (11), representing a membranous and cellular gateway that must be traversed for SPZs to gain access to the next host. Studies of mosquito SG biology to date have emphasized morphological descriptions by electron microscopy (EM) (12–16), identification of saliva components (17), or investigation of surface proteins and other molecules (18–20) that contribute to sporozoite invasion. Interestingly, SG cell death can limit disease transmission indirectly through an effect on parasite development in the midgut (21). Sporozoite ejection has been visualized by live imaging (22) and was found to occur even with sugar feeding (23).

Prior studies have noted the disparity between the very high numbers of SPZs found within mosquito SGs and the much smaller number ejected into the mammalian host (22, 24, 25). Numbers of SG SPZs range widely among field populations (26). Our previous studies (surveying gland-to-gland variations in SG architecture, determining how the adult SG forms the secretory portion of the salivary duct, and describing how SG secretory cells acquire their unusual cup-shaped morphology [27, 28]) suggested that cell architecture variation may play a role in the journey of sporozoites through the SGs. Prior electron microscopy analysis included limited sample sizes and did not allow visualization of multiple molecular markers. Therefore, we applied immunofluorescence (IF) confocal microscopy to hundreds of infected mosquito SGs to study SPZ localization and SG features at multiple infection intensities. We found that several SG architectural features create barriers to SPZ traversal.

## RESULTS

### Sporozoite localization within infected salivary glands.

**FIG 1:**
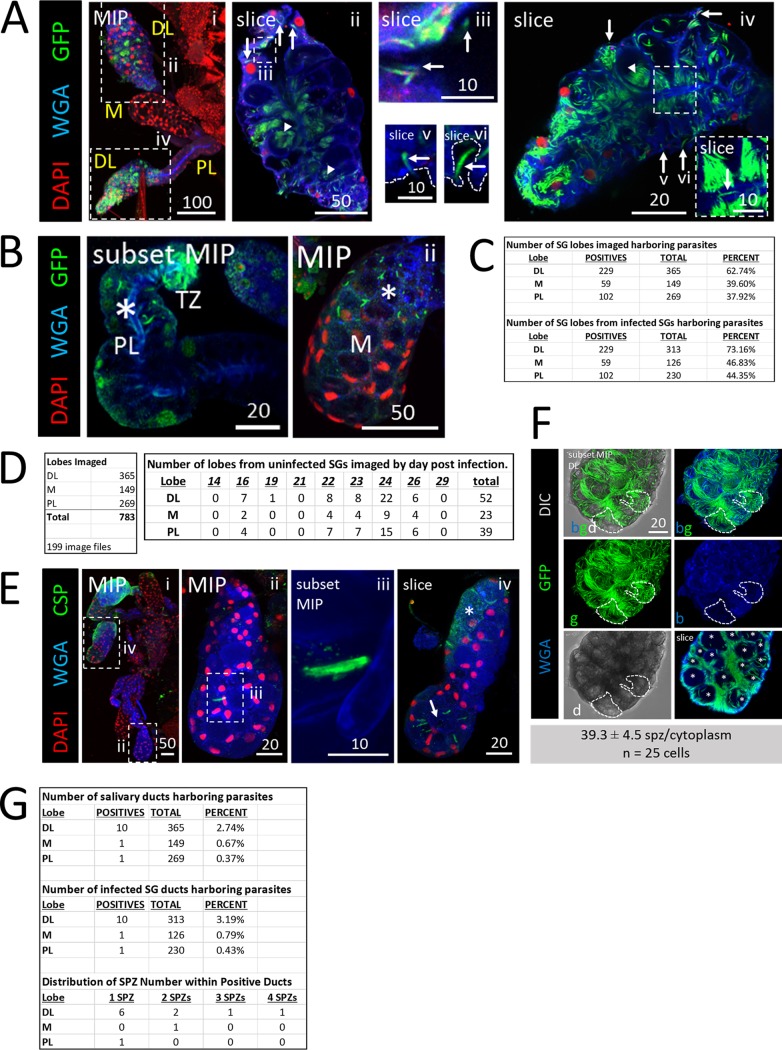
*Plasmodium* sporozoites (SPZs) primarily invade the distal lateral lobes. Representative images of the entire depth (maximum intensity projection [MIP]) or partial depth (subset MIP) or single-slice confocal images of salivary glands (SGs) stained with DAPI (DNA, red), WGA (chitin/O-GlcNAcylation, blue), and either GFP (panels A and C to D; SPZs, green) or CSP (panel B; a SPZ protein, green) 18 to 24 days postinfection with P. berghei. Scale bar length units are micrometers. (A) Low and high magnification of SG with only the distal lobes infected and showing where SPZs were found: in secretory cell cytoplasms (panel ii, arrows), in secretory cavities (panel ii, arrowheads), in large, central, fluid-filled lumens (panel iii, arrows), in locations associated with the basement membrane (panel iv-vi, arrows), and rarely, inside the salivary duct (panel iv inset, arrow). The contrast in the inset in panel iv was uniformly enhanced to highlight the salivary duct SPZ and in panels v to vi to highlight the SPZs and secretory cell cytoplasms. The basement membrane (from the DIC channel; not shown) is marked by a dashed line (panels v and vi). (B) Representative images of PL (panel i) and M (panel ii) lobe infections. Multiple SPZs are observable (asterisks). (C) Number of lobes of different types imaged in this study (top) and number of SG lobes harboring parasites out of total infected SGs (bottom). (D) Total lobes imaged (left) and number of lobes imaged from uninfected SGs (right). (E) SG SPZ numbers differed from lobe to lobe, even within a single mosquito. A single SPZ was observed in one DL lobe (panel ii), in proximity to a fused salivary duct (panel iii). In another DL lobe from that mosquito, about 10 SPZs were oriented toward an irregular, round lumen (panel iv, arrow). Some infected lobe regions contained accumulations of what was likely shed CSP (panel iv, asterisk). (F) Representative image from a SG with secretory cell cytoplasms filled with SPZs (see the full lobe image in Fig. 4A and the description in the corresponding figure legend) that was used to determine that ∼40 sporozoites can occupy the cytoplasmic volume of a typical SG secretory cell (*n* = 25 cells). In this lobe, SPZs inside secretory cavities (slice image, asterisks) were easily discernible and were excluded from cytoplasmic SPZ counts. Two cells are outlined in white dashes. (G) Frequency and distribution of SPZs observed inside the salivary duct.

We dissected, stained, and imaged Anopheles stephensi salivary glands (SGs) between 14 and 29 days postinfection with one of several strains of Plasmodium berghei (see Materials and Methods) to determine sporozoite (SPZ) localization (Fig. 1). Distal lateral (DL) lobes were the primary site of SPZ occupancy (Fig. 1A, panels i, ii, and iv). SPZs were observed in association with the basement membrane (Fig. 1A, panels ii and iv to vi), pockets of saliva between the basement membrane and secretory cells (Fig. 1A, panel iv), inside secretory cell cytoplasms (Fig. 1A, panels iii and iv), in secretory cavities (Fig. 1A, panels ii and iv), next to the salivary duct (Fig. 1A, panel iv), inside the SG lumen (Fig. 1A, panel iii), and in the salivary duct (Fig. 1A, panel iv [inset]). Notably, SPZs were frequently seen in proximal lateral (PL) lobes (Fig. 1B, panel i, asterisk) and medial (M) lobes (Fig. 1B, panel ii, asterisk). In 783 SG lobes (365 DL, 149 M, and 269 PL) imaged (Fig. 1D), SPZs were observed in about 73% of DL lobes, 47% of PL lobes, and 44% of M lobes from infected SGs (Fig. 1C). Among the imaged SGs, 52 DL, 23 M, and 39 PL lobes were from SGs that completely lacked SPZs (Fig. 1D). Numbers of SPZs differed between SGs and lobes of the same mosquito (Fig. 1E). In one DL lobe, a single SPZ was seen near a fused shut salivary duct (Fig. 1E, panels ii and iii); in the other DL lobe of the same mosquito, about 15 SPZs were present (Fig. 1E, panel iv). In another DL lobe, secretory cell cytoplasms (Fig. 1F, slice), but not secretory cavities (Fig. 1F slice, asterisks), were packed full of SPZs. Counts in this lobe revealed that approximately 40 SPZs can fit within a single DL lobe secretory cell cytoplasm (Fig. 1F). SPZs were rarely observed within the salivary duct and only in small quantities (Fig. 1G).

**FIG 2:**
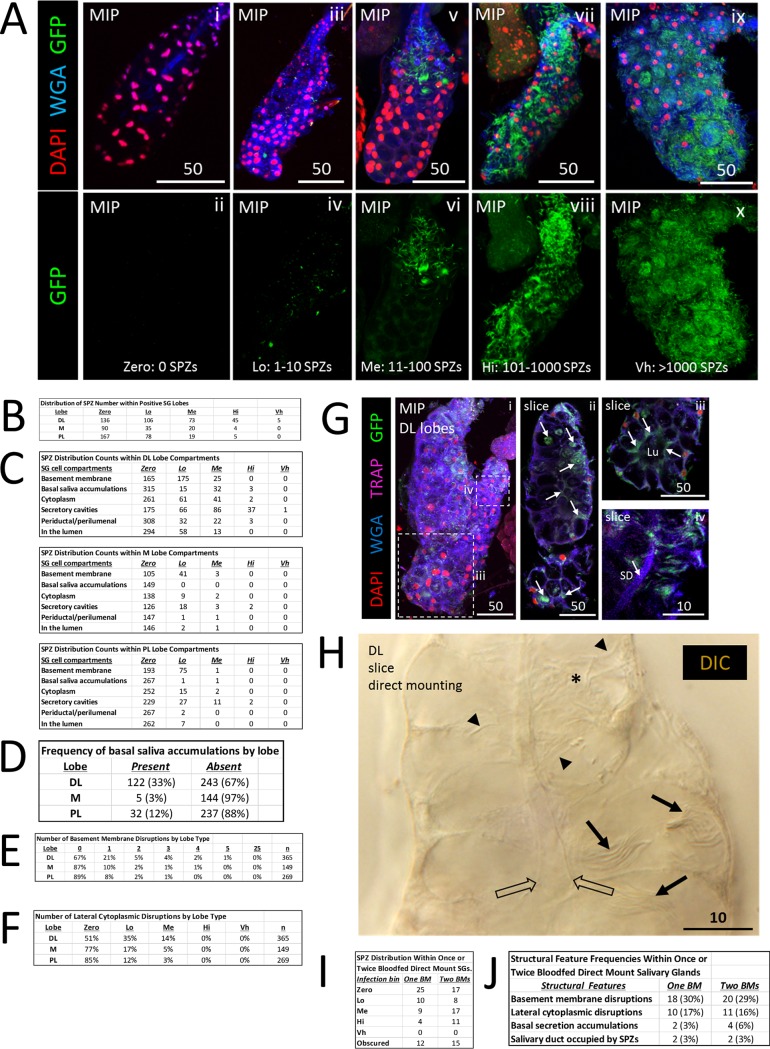
Quantification of salivary gland infection by *Plasmodium* sporozoites reveals invasion tendencies. (A) Examples of SGs with different numbers of invaded SPZs used for binning indicated as follows: no SPZs—Zero: 0 SPZ (panels i and ii); low numbers of SPZs—Lo: 1–10 SPZ (panels iii and iv); medium numbers of SPZs—Me: 11–100 SPZ (panels v and vi); high numbers of SPZs—Hi: 101–1000 SPZ (panels vii and viii); and very high numbers of SPZs—Vh: >1000 SPZ (panels ix and x). (B) Distribution of SPZ numbers in different lobes from infected SGs. (C) Localization of SPZs in the different lobes at different infection levels. At lower infection levels, most of the SPZs were associated with the basement membrane. At higher infection levels, most of the SPZs were found in secretory cavities. (D) Frequency of basal saliva accumulation by lobe in infected glands. (E) Frequency of basement membrane disruptions in different lobes from infected glands. (F) Frequency of lateral cytoplasmic disruptions in the different lobes at different infection levels. (G) Following a second, noninfective blood meal (BM) 23 days after P. berghei infection, SPZs were observed in similar SG locations: in secretory cell cytoplasms, in secretory cavities, and in the lumen (panels ii and iii, arrows). No increase in salivary duct occupancy was observed with greater SPZ numbers or with a second blood meal (panels G [iv] and C). Signal contrast was uniformly increased in panel G (iv) to highlight the salivary duct (SD). (H) In parallel, the infected mosquitoes in a second cage were given a second blood meal, and then female SGs were dissected and directly mounted on slides. Results showed individual (arrowheads) and bundled (arrows) sporozoites in similar numbers (quantified in panel I) and locations (secretory cell cytoplasm, secretory cavities, and lumen) and similar SG structural abnormality frequencies (quantified in panel J).

To better address SG localizations or structural features associated with a variety of SPZ numbers, we scored each lobe independently and assigned SG lobes into bins based on the number of SPZs present (Fig. 2A and C) as follows: Zero, 0 SPZs; Lo (low numbers of SPZs), 1 to 10 SPZs; Me (medium), 11 to 100 SPZs; Hi (high), 101 to 1,000 SPZs; Vh (very high), >1,000 SPZs. Lobes with lower numbers of SPZs were more prevalent than those with higher numbers (Fig. 2B). In lobes with low quantities, SPZs were most frequently observed in association with the basement membrane; in lobes with higher quantities, SPZs were most frequently seen in the secretory cavities (Fig. 2C). We counted several types of structural and morphological defects. Saliva accumulations between secretory cells and the basement membrane (Fig. 2D), basement membrane disruptions (Fig. 2E), and secretory cell cytoplasmic disruptions (Fig. 2F) were each observed most frequently in the DL lobes.

The low frequencies and numbers of SPZs seen in the salivary duct were unexpected, so we asked if providing a second blood meal to previously infected mosquitoes at a time of high SPZ occupancy (day 23 postinfection) of SGs would alter SPZ localization (Fig. 2G). Surprisingly, we did not observe any differences in SPZ localization. SPZs were seen at the basement membrane, in secretory cell cytoplasms, and in secretory cavities (Fig. 2G, arrows). A small increase in the levels of Me and Hi infection was seen after a second blood meal (Fig. 2I), consistent with their 2-h wait compared to paired “no second blood meal” mosquitoes. Similar rates of SG structural defects and salivary duct SPZ occupancy were observed regardless of whether or not a second blood meal was provided (Fig. 2J). Importantly, no increase in salivary duct occupancy by SPZs was seen after the second blood meal.

We also considered trends in the relationships between time postinfection, SPZ organization (individuals versus bundles), infection intensity, and structural defects in our data (see Fig. S1A to E in the supplemental material). Infection intensity peaked at days 23 to 24 postinfection (Fig. S1A), in line with Johns Hopkins Bloomberg School of Public Health Malaria Research Institute (JHMRI) Insectary condition estimates (29). Individualized SPZs were more prevalent than bundled SPZs, and the numbers of both peaked with age (Fig. S1B) and high infection intensity (Fig. S1C). Higher infection intensity was associated with more occurrences of basement membrane disruptions (Fig. S1D) and secretory cell cytoplasmic disruptions (Fig. S1E) in all lobe types.

10.1128/mBio.01238-19.1 FIG S1 Multivariable comparisons of salivary gland infection data. Tables display comparisons of two or three variable breakdowns representing age, structural, or localization factors that may relate to sporozoite (SPZ) invasion phenotypes. Data are broken down into the period before SG infection (days 14 to 21 postinfection [p.i.]), the period of peak SG infection (days 22, 23, and 24 p.i.), and the period after peak SG infection (days 26 or 29 p.i.) as needed. Sample sizes are listed in “n” columns. (A) SPZ counts per lobe based on day postinfection. (B) Distributions of individual and bundled SPZs per lobe based on day postinfection. (C) Distributions of individual and bundled SPZs per lobe based on infection intensity. (D) Frequency of basement membrane disruptions per lobe based on infection intensity. (E) Frequency of cells with lateral cytoplasm disruptions per lobe based on infection intensity. (F) SG lobe length and width based on day postinfection. (G) SG lobe length and width based on level of infection. (H) Comparison of SG lobe dimensions from all samples either directly mounted (pink) or processed for immunofluorescence confocal microscopy (green). Error bars shown represent 1 standard deviation from the mean. Asterisks denote significant differences in length and width (2-sample *t* test, *P* < 0.05). Sample sizes (from left to right) were 120, 365, 120, 365, 60, 149, 60, 149, 120, 269, 120, and 269. (I) Paired-value scatter plot of lobe length versus width across all lobe types (DL [blue], M [orange], and PL [gray]) from immunostained SGs. (J) Paired-value scatter plot of lobe length versus width for directly mounted SGs from mosquitoes given one infective blood meal. The data suggest that directly mounted SG dimensions have more consistent lobe dimensions. Download FIG S1, TIF file, 0.9 MB. Copyright © 2019 Wells and Andrew. 2019 Wells and Andrew This content is distributed under the terms of the Creative Commons Attribution 4.0 International license.

Finally, we interrogated the effects of processing and imaging SG tissue samples for immunofluorescence on SG structure and SPZ localization using differential interference contrast (DIC) imaging (Fig. 2H). SPZs were visible as individuals (Fig. 2H, arrowheads) or in groups (Fig. 2H, arrows), with the highest numbers of SPZs seen in the secretory cavities. No SPZs were observed in the salivary ducts.

### CSP localization during salivary gland invasion and egress.

**FIG 3:**
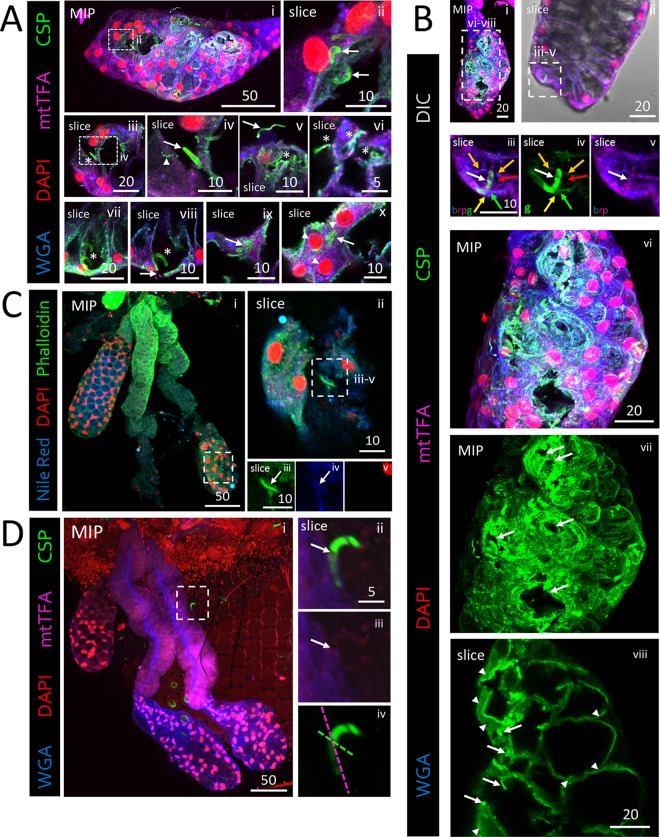
Salivary gland basement membrane and secretory cell traversal by sporozoites (SPZs) is associated with changes in the invasion and motility protein CSP. Representative 3D projection (MIP) or single-slice (slice) confocal microscopy images from salivary glands (SGs) stained with DAPI (DNA, red), either WGA (A, B, and D; chitin [O-GlcNAcylation], blue) or Nile red (C; lipids, blue), and either antisera against CSP (A, B, and D; SPZ protein, green) and mtTFA (A, B, and D; mitochondria, purple) or phalloidin (C; actin, green) 22 (A, B, and D) or 24 (C) days postinfection with P. berghei are shown. Scale bar length units are micrometers. (A) Distal lateral lobe showing large basement membrane disruptions (panel i) and rounded, likely dead parasites (panel ii). The examples shown represent cellular invasions and egressions by SPZs (panels iii to ix, asterisks). Three distinct CSP morphologies were observed: thick CSP SPZ coat (panel iv, arrow), thin CSP SPZ coat (panel v, arrow), and smaller tracks of shed CSP (panel iv, arrowhead). Thin CSP-coated SPZs are shown in secretory cavities (panel vi, asterisks). Thick CSP-coated parasites were observed invading secretory cells through the secretory cavities (panels vii and viii, asterisks) with a nearby basement membrane/cell disruption (panel viii, arrow). Rare SPZs with thick CSP coats were observed within damaged, dysmorphic SG secretory cell cytoplasms (panels ix and x, arrows). Perinuclear mtTFA enrichment in invaded cells is indicated (panel x, asterisks). (B) Low-magnification and high-magnification images of an infected distal lobe. A single SPZ (panel iii, white arrow) captured crossing the SG basement membrane (panels iii and iv, yellow arrow), a secretory cell cytoplasm (panels iii and iv, green arrow), a secretory cavity (panels iii and iv, red arrow), and lateral cytoplasmic extensions, which define the sides of the secretory cavity, of that cell and a neighboring cell (panels iii and iv, orange arrows) is shown. Contrast was uniformly enhanced in panel iv to highlight CSP and the SG cellular contents (WGA, mtTFA) associated with the invading sporozoite (panel v, white arrow) extending into the secretory cavity. Large basement membrane disruptions and large accumulations of shed CSP are shown (panels vi and vii, arrows). SPZs (panel viii, arrows) and CSP accumulations (panel viii, arrowheads) are observable. (C) Low-magnification image of a DL (panel i) with a SPZ (panels ii to iv) inside the lumen associated with a molecular halo (panels iii and iv, white arrow) that included Nile red and phalloidin staining accumulations. (D) Low-magnification image of SGs with only a few surrounding SPZs (panel i). Images of a single SPZ during invasion show that CSP staining was highly reduced beginning at the likely point of invasion (panels ii and iii, arrow). The basement membrane (purple dashed line) and plane of invasion (green dashed line) are indicated (panel iv).

To better understand SPZ ingression and egression events in SGs, we stained infected SGs with an antibody that recognizes both processed and nonprocessed forms of circumsporozoite protein (CSP). CSP is the major protein present on the SPZ coat, is involved in motility, and may promote SG invasion (16, 30–33). We observed three CSP staining patterns: SPZs with a thick CSP coat (Fig. 3A, panel iv, arrow), SPZs with a thin CSP coat (Fig. 3A, panel v, arrow), and tracks likely composed of shed CSP within cells (Fig. 3A, panel iv, arrowhead). These data support prior indications that CSP coat morphology correlates with SG invasion status (31). A thick CSP coat is present on SPZs that have not invaded a SG cell, whereas a thin CSP coat indicates SPZs that have invaded a SG cell. Infrequently, instances of thick CSP-coated SPZs were observed in secretory cell cytoplasms (Fig. 3A, panels ix and x). Strong accumulations of CSP signal were observed in lobes containing basement membrane and cellular disruptions (Fig. 3B, panel vii). Sometimes, both SPZ coat-associated CSP (arrows) and shed CSP (arrowheads) were present (Fig. 3B, panel viii). Secretory cell cytoplasmic markers were regularly found in association with the coat of some SPZs after invasion (Fig. 3B, panel v, and C). These results suggest that the CSP-containing coat is largely shed as SPZs invade secretory cells but that it may be retained when SPZs enter through a large disruption. Accordingly, CSP staining (16, 30, 31) was greatly reduced internally to the basement membrane along a single SPZ during invasion (Fig. 3D; see also Movie S1). Within SG secretory cells, we observed two sites of shed CSP accumulation (Fig. S2): at the apical surface (Fig. 3D, panel viii; see also Fig. S2C to H, yellow arrows) and in a more basal, cytoplasmic compartment showing relative depletion of cellular markers (Fig. S2D to I, white arrows) (mitochondrial transcription factor A [mtTFA], purple; wheat germ agglutinin [WGA], blue). These results suggest that CSP shedding continues during SG secretory cell invasion, traversal, and egress.

10.1128/mBio.01238-19.2 FIG S2 Invaded salivary gland secretory cells contain shed sporozoite (SPZ) CSP. A representative maximum intensity projection (MIP) image and slice confocal images from a DL lobe stained with DAPI (DNA, red), WGA (chitin [O-GlcNAcylation], blue), and antisera against mtTFA (mitochondria; purple) and CSP (SPZ protein, green) 22 days after infection with P. berghei are shown. (A) CSP accumulates at basement membrane disruptions near sites of invasion (yellow arrows). (B to H) CSP was observed in secretory cells throughout the cytoplasm (B) and was enriched at two sites: (i) at or near the apical surface (C to H, yellow arrow) and (ii) more basally, in regions with low levels of WGA and mtTFA staining (C to H, white arrows). (C and I) Line scan analysis (C and I, white dashed line) showing relative enrichment of CSP signal in areas of relative mtTFA and WGA signal depletion (I, red dashed lines). Scale bar length units are micrometers. Download FIG S2, TIF file, 2.8 MB. Copyright © 2019 Wells and Andrew. 2019 Wells and Andrew This content is distributed under the terms of the Creative Commons Attribution 4.0 International license.

10.1128/mBio.01238-19.5 MOVIE S1 Sporozoite traversal of salivary gland basement membrane, secretory cells, and secretory cavity. Bar, 5 μm. This movie corresponds to panel iv of Fig. 3B and shows the Z-stack frame-by-frame progression (1-μm step size) of a single SPZ that had almost completely crossed the salivary gland DL lobe basement membrane, traversed a secretory cell cytoplasm and the associated secretory cavity and the cytoplasm again, and breached the neighboring secretory cell. The sample was stained with the dyes WGA (chitin [O-GlcNAcylation], blue) and DAPI (DNA, red) and with antisera against mtTFA (not shown) (mitochondria, purple) and CSP (SPZ marker, green). Download Movie S1, AVI file, 0.1 MB. Copyright © 2019 Wells and Andrew. 2019 Wells and Andrew This content is distributed under the terms of the Creative Commons Attribution 4.0 International license.

### Sporozoites accumulate at physical barriers.

**FIG 4:**
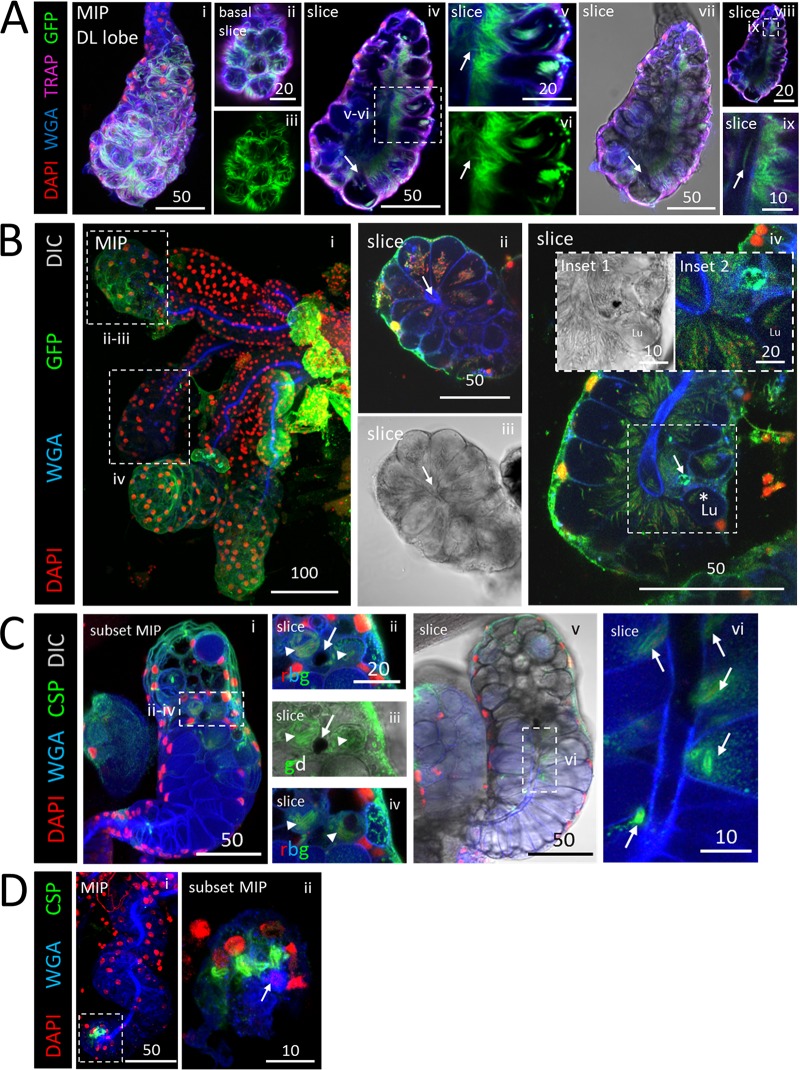
Salivary gland architectural features associated with sporozoite (SPZ) accumulation. Images represent 3D projections over the entire salivary gland (SG) depth (MIP) or partial SG depth (subset MIP) or single-slice (slice) images from salivary glands stained with DAPI (DNA, red), WGA (chitin [O-GlcNAcylation], blue), and either GFP [A and C; SPZs, green] or CSP (B; SPZ protein, green) 23 (A and C) or 24 (B) days postinfection with P. berghei. Scale bar length units are micrometers. (A) SPZs near the basal surface of this DL lobe were individualized (panels i to iii), whereas SPZs in the apical region were tightly packed and oriented toward the salivary duct and lumen across much of the gland (panels iv and vii to ix). One secretory cavity containing SPZs was open to the lumen (panel iv, arrow). A single SPZ traversing a disruption in the salivary duct wall (panel v, vi, arrow) and a SPZ inside the salivary duct (panel ix, arrow) are shown. (B) Low-magnification image of infected glands (panel i). A fused duct terminus with sporozoites (SPZs) grouped in adjacent secretory cavities is shown in panels ii and iii. A DL lobe with a thick-walled salivary duct and swollen duct terminus connected to a small, mispositioned lumen with SPZs clustered at the duct wall and largely oriented toward the duct is shown in panel iv and in inset 1 in that panel. A possible passage, enriched for GFP, connecting the lumen to the adjacent secretory cavity is shown in panels iv (arrow) and iv (inset 2). SPZs were observed inside the lumen (panel iv, asterisk). (C) DL lobe with basement membrane and cell disruption (panels ii to iv, arrow) with two adjacent compartments filled with thick CSP-coated SPZs (panels ii to iv, arrowheads) in the same z focal plane (panel ii and iii) and in a different z focal plane (panel iv). More distally located secretory cells were open to the lumen (panel v), whereas more proximally located secretory cells had no periductal space and clustered SPZs near the duct (panel vi, arrows). (D) A corkscrewing SG with no lumen and a fused duct terminus (panel i) contained thick CSP-coated SPZs nearby (panel ii, arrow).

SPZs accumulated at sites of physical barriers within the SGs (Fig. 4A). The most notable obstruction was the salivary duct wall within proximal secretory cells of the DL lobe. SPZs were frequently observed oriented perpendicularly to the thick chitinous secretory duct wall, which extends about halfway into DL lobes, just interior to the secretory cavities. A periductal space is visible between the apical edge of secretory cells and the salivary duct when sufficient saliva is present. Near the basal surface of this DL lobe (Fig. 4A, panels ii and iii), SPZs were mainly individualized and randomly oriented. Internally, this lobe showed a defect in the usually open interface between most secretory cavities and the lumen (Fig. 4A, panels iv and vi). In this example, the chitinous duct appeared to extend around the outer lumenal interface, dividing the lumenal and secretory cavity territories in all but one DL lobe cell (Fig. 4A, panel iv, arrow). SPZs were lined up perpendicularly to this extended chitinous boundary (Fig. 4A, panels v and ix). One SPZ was seen crossing the boundary through a small disruption (Fig. 4A, panels v to vii, arrows), and a SPZ was observed nearby inside the salivary duct (Fig. 4A, panel ix). We found additional examples of SG cell architectures acting as barriers to SPZ traversal. One contained two barrier architectures (Fig. 4B). In one DL lobe, the salivary duct terminus was fused shut (Fig. 4B, panel ii, arrow). SPZs filled the secretory cavities (Fig. 4B, panels ii and iii), but no SPZs were observed inside the duct. In another DL lobe, a widened, thickened salivary duct was seen to connect to a small, round, mispositioned lumen (Fig. 4B, panel iv [inset 1, Lu]). SPZs were seen in secretory cavities aligned perpendicularly to the duct (Fig. 4B, panel iv). SPZs were present in the lumen (Fig. 4B, panel iv, asterisk), which might have been made accessible by a secretory cell disruption enriched for SPZ green fluorescent protein (GFP) (Fig. 4B, panel iv, arrow; see also panel iv, inset 2), forming a lumenal-periductal space passage. One DL lobe (Fig. 4C) contained a circular disruption in the middle of the lumen at the salivary duct terminus (Fig. 4C, panels i and vi) which blocked SPZ traversal from secretory cells to the lumen and/or from the lumen to the duct (Fig. 4C, panels ii to iv). WGA signal, likely from secreted O-GlcNAcylated proteins, was enriched proximally to the disruption within the SG (Fig. 4C, panel v). SPZs did not traverse the salivary duct wall in the proximal region (Fig. 4C, panel vi). Finally, one DL lobe (Fig. 4D) was corkscrewed and contained a fused salivary duct near the basal tip of the lumen (Fig. 4D, panel ii, arrow). SPZs were clustered around, but not found inside, the fused duct. These data indicate that SG architectural features can hinder SPZ invasion and egress.

### Salivary gland morphology, cell damage, and secretion consequences from infection.

To look for effects of SPZ invasion on SG morphometry, we measured SG lobe maximal width and maximal length under various conditions. In general, lengths and widths were similar across days postinfection and levels of infection intensity for all three lobe types (Fig. S1F and G). Small differences were seen in DL lobe length, DL lobe width, and PL lobe width in comparisons of SGs processed for immunofluorescence to directly mounted samples (Fig. S1H). To explore this further, we created scatter plots mapping each lobe’s length and width during processing for immunofluorescence (Fig. S1I) or direct mounting (Fig. S1J). Strikingly, the directly mounted samples had much more consistent lengths and widths for all three lobes than the samples processed for IF (Fig. S1I and J). This suggests that the major effect of our 90-s acetone permeabilization for antibody staining was a weakening of the SG basement membrane that allowed SGs to splay more randomly when the coverslip was placed during mounting. No evidence of tissue shrinking during sample processing for staining was observed. Thus, we found that SG invasion had little impact on overall SG morphology.

**FIG 5:**
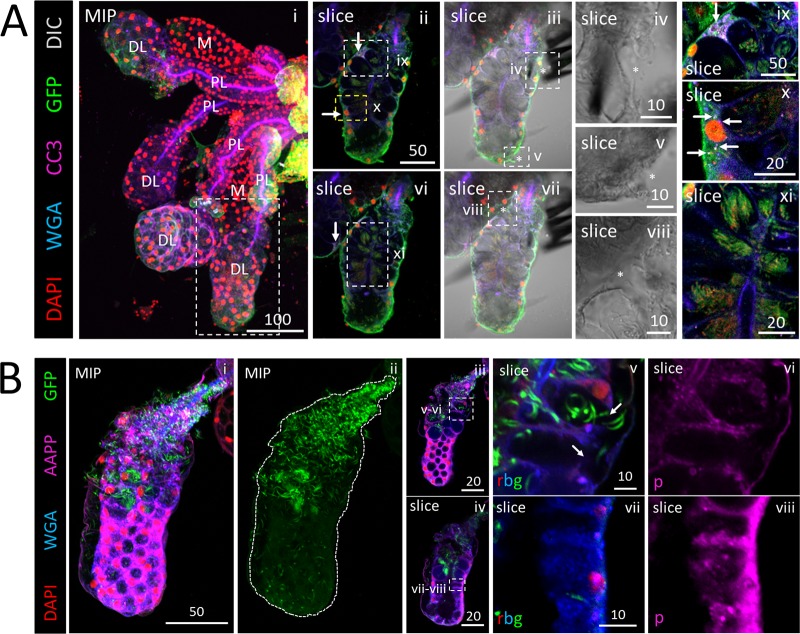
Apoptosis accompanying moderate invasion can be minimal, whereas large numbers of sporozoites (SPZs) can disrupt cell structure and saliva protein signal. (A and B) 3D projection (MIP) or single-slice confocal images of a representative distal lateral (DL) lobe stained with DAPI (nuclei, red), WGA (chitin [O-GlcNAcylation], blue), and antisera against GFP (SPZs, green) and either cleaved caspase 3 (A; CC3, purple) or the saliva protein *Anopheles* antiplatelet protein (B; AAPP, purple) 23 (A) or 24 (B) days postinfection with P. berghei. Scale bar length units are micrometers. (A) A distal lobe with large numbers of SPZs (panels ii, vi, and xi) had only two cells with accumulations of the apoptosis marker CC3 (panels ii, ix, and x, arrows) and only three small basement membrane disruptions (panels iii to v and vii to viii, asterisks). The images in panels ii to v and vi to viii are from two different focal planes. A neighboring DL lobe with a single CC3-positive cell is shown in panel vi (white arrow). Signal contrast was uniformly enhanced in panels ix to xi to highlight CC3 signal (panels ix and x) and SPZs (panel xi). (B) A DL lobe (panel i) with greatly different numbers of SPZs in the proximal and distal regions (panel ii). High SPZ numbers in the proximal region correlated with cell disruption (panel v, arrows [split secretory cell distal cytoplasms and lost basement membrane attachment]) and greatly reduced levels of AAPP saliva protein staining (panel v) compared to the distal region (panel vii).

We assessed the extent of SG morphological alteration with SPZ invasion. We found examples of robustly invaded SGs with nearly unperturbed structures and very little evidence of cell death, as well as examples of SGs with many disrupted cells and decreased saliva protein abundance (Fig. 5). For example, one DL lobe (Fig. 5A) contained many secretory cavity SPZs (Fig. 5A, panels vi and xi) but had only three small basement membrane disruptions (Fig. 5A, panels iv, v, and viii) and two cells positive for the apoptosis marker cleaved caspase 3 (CC3, purple; Fig. 5A, panels ix and x). These data indicate that extensive SG invasion by SPZs in the absence of large numbers of dying cells can occur, as previously suggested (16). Another sample DL lobe (Fig. 5B, panel i) contained many SPZs in the proximal portion but many fewer in the distal portion (Fig. 5B, panel ii). In the proximal DL lobe, secretory cell cytoplasms were disrupted (Fig. 5B, panel v, arrows), saliva accumulated basally, and SPZs were observed in the basal saliva (Fig. 5B, panel v), whereas the typical cup shape of secretory cells in close association with the basement membrane was preserved in the distal DL lobe (Fig. 5B, panel vii). Staining for *Anopheles* antiplatelet protein (AAPP, purple) was much weaker in the disrupted proximal DL lobe (Fig. 5B, panel vi) than in the intact distal DL lobe (Fig. 5B, panel viii). These data suggest that some invaded SGs sustain extensive cellular damage that reduces secretory protein abundance.

### Sporozoite interactions inside salivary glands.

**FIG 6:**
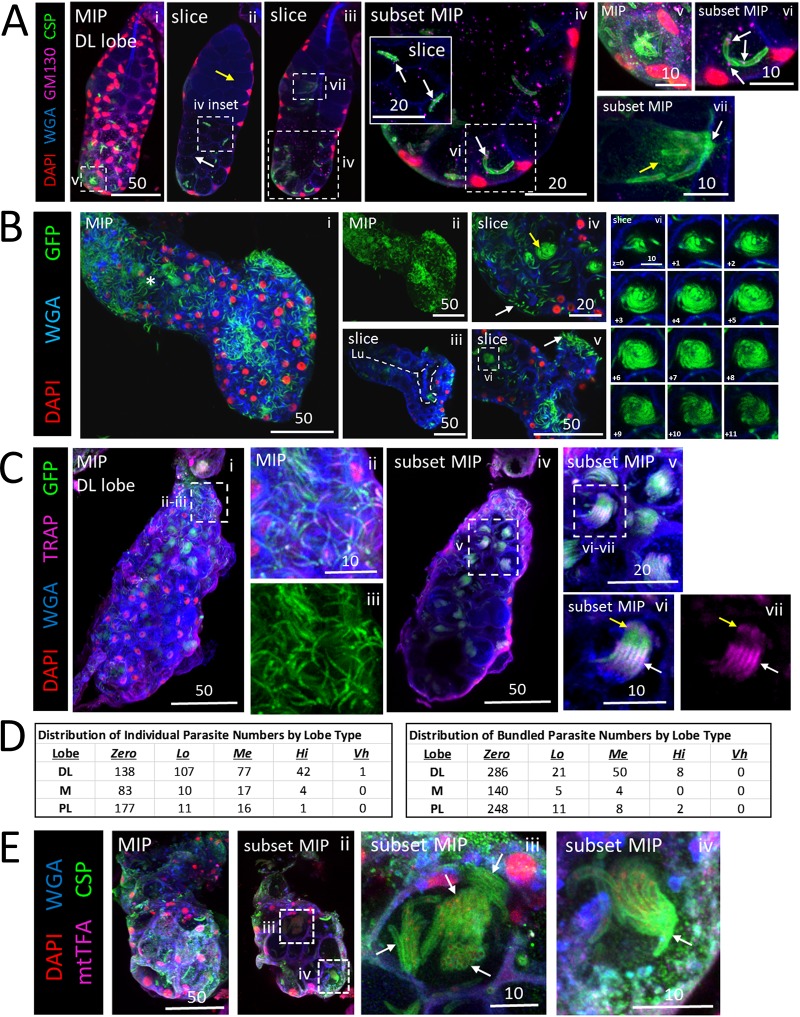
Polarized organization of sporozoites (SPZs) within bundles. (A to D) 3D projection across entire SG depth (MIP) or part of the SG depth (subset MIP) images of a representative distal lateral (DL) lobe stained with DAPI (nuclear, red), WGA (chitin [O-GlcNAcylation], blue), and antisera against either GM130 (Golgi/cytoplasm, purple) and CSP (SPZ protein, green) (A), CSP alone (SPZ protein, green) (B), TRAP (SPZ protein, purple) and GFP (SPZs, green) (C), or mtTFA (SG cell cytoplasm, purple) and CSP (SPZ protein, green) (D) either 24 (A and B), 23 (C), or 22 (D) days postinfection with P. berghei. Scale bar length units are micrometers. (A) A DL lobe with only few SPZs in the proximal portion (panel ii, yellow arrow) and with more in the distal portion (panel ii, white arrow). Individual SPZs were observed (panel iv, inset) as well as SPZs interacting in a variety of quantities, including two (panels iv and vi), six or eight (panels iii and vii), or 10 to 12 (panels i and v). Note the concentrated shed CSP (panel vii, yellow arrow) at the salivary duct wall (panel vii, white arrow). GM130 was present in close proximity to the CSP coat of a SPZ in the secretory cavity (panels iv [and inset] and vi [white arrows]). (B) A bulbous DL lobe with high numbers of SPZs (panels I and ii) had an irregularly shaped lumen (panel iii, white dashed line). The asterisk in panel i marks the location of the salivary duct terminus. SPZs occupied the space between the basement membrane and the secretory cells (panels iv and v, white arrow), and SPZ groups were seen in a circling/swirling pattern (panels iv [yellow arrow] and vi [arrows]; see Movies S2 and S3). In panel vi, relative z positions are given in micrometers. (C) Infected DL lobe with both individualized SPZs (panels ii and iii) and SPZ bundles (panels iv to vii). TRAP localization (purple) to the apical tip (panels vi and vii, yellow arrows) and medial (panels vi and vii, white arrows) regions of SPZs (panels vi and vii) indicates that all SPZs within a bundle are similarly oriented anterior to posterior. (D) Frequencies of individual (left) or bundled (right) SPZs by lobe type. (E) Multiple SPZ bundles in a single secretory cavity (panel iii, arrows), with enrichment of CSP observable at basal end of a sporozoite bundle (panel iv, arrow), at a site of basement membrane disruption (panels i and ii, dashed boxes). CSP contrast was uniformly enhanced in panel iii to highlight the SPZs.

We observed several types of SPZ interactions within SGs beyond bundling (Fig. 6A). SPZs were found in contact in a variety of quantities, from 2 SPZs (Fig. 6A, panel vi) to 6 to 8 (Fig. 6A, panel vii) or 10 to 12 (Fig. 6A, panel v). In several SGs, we observed groups of SPZs with circling/spiraling organization within secretory cavities (Fig. 6B, panel iv, yellow arrow, and panels v to vi; see also Movies S2 and S3 in the supplemental material). The role of circling/spiraling in SPZ biology is currently unknown. Often, both individualized SPZs (Fig. 6C, panels ii and iii) and bundled SPZs (Fig. 6C, panels iv to vii) were found within the same DL lobe. Individualized SPZs were observed more frequently and in greater numbers than bundled SPZs (Fig. 6D). Whereas individualized SPZs were oriented randomly, as revealed by the SPZ GFP signal (Fig. 6C, panel iii), staining for the *Plasmodium* thrombospondin repeat-associated protein (TRAP), a motility and invasion protein (34, 35), demonstrated that all SPZs within a bundle were oriented in the same direction (Fig. 6C, panel vii). We also observed an example of multiple, differentially oriented bundles within a single secretory cavity (Fig. 6E, panel iii, arrows). A bundle of SPZs, elsewhere in this lobe, enriched at one end for CSP signal (Fig. 6E, panel iv, arrow) was observed within a basement membrane disruption. These observations raise the possibility that bundling may allow SPZs to pool their enzymatic and/or mechanical efforts to move through physical barriers.

10.1128/mBio.01238-19.6 MOVIE S2 Circling/spiraling SPZs inside a DL lobe. Bar, 20 μm. This movie relates to panel v of Fig. 6B and shows a Z-stack frame-by-frame progression (1-μm step size) of several groups of circling/spiraling SPZs inside a DL lobe. The sample was stained with the dyes DAPI (DNA, red) and WGA (chitin [O-GlcNAcylation], blue) and with antisera against GFP (SPZ marker, green). Download Movie S2, AVI file, 0.2 MB. Copyright © 2019 Wells and Andrew. 2019 Wells and Andrew This content is distributed under the terms of the Creative Commons Attribution 4.0 International license.

10.1128/mBio.01238-19.7 MOVIE S3 Numerous groups of circling/spiraling SPZs inside a heavily infected DL lobe. Bar, 20 μm. This movie relates to panel x of Fig. 2A and shows a Z-stack frame-by-frame progression (1-μm step size) of many groups of circling/swirling SPZs. The sample was stained with DAPI (not shown) (DNA, blue), WGA (not shown) (chitin [O-GlcNAcylation], red), and GFP (SPZ marker; green). Download Movie S3, AVI file, 1.3 MB. Copyright © 2019 Wells and Andrew. 2019 Wells and Andrew This content is distributed under the terms of the Creative Commons Attribution 4.0 International license.

## DISCUSSION

**FIG 7:**
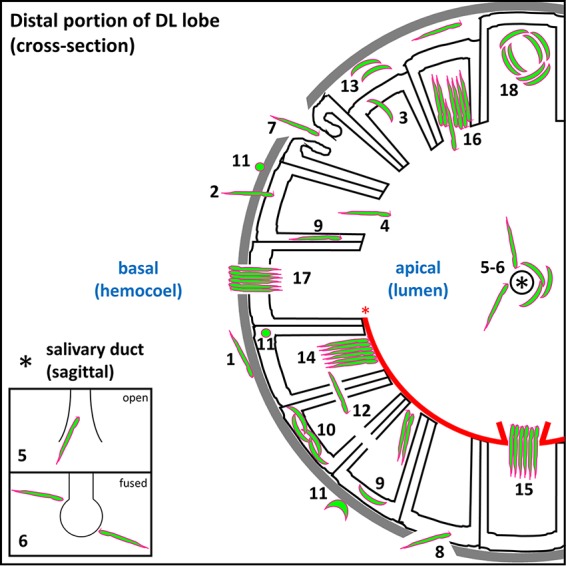
Roadblocks along the journey of *Plasmodium* sporozoites (SPZ) through *Anopheles* mosquito salivary glands. Schematic diagram depicting a cross-sectional view of a distal lateral (DL) lobe, infected by SPZs that encountered every barrier to salivary gland (SG) entry and exit observed in this study. Apical (SG lumen) and basal (mosquito hemocoel) compartments are noted (blue text). SPZs must interact with (step 1) and traverse (step 2) the basement membrane (gray), enter (step 2) and exit (step 3) the secretory cell cytoplasm (outlined in black) into the secretory cavity, enter the lumen (step 4), and finally enter the salivary duct (step 5) (the asterisk indicates the duct lumen) to proceed out of the mosquito during the next blood meal. Salivary ducts were sometimes (Fig. 1E, panel iii; see also Fig. 4B, panel ii, and D, panel ii) fused shut (step 6), as seen previously in male salivary glands (27, 28). Some lobes developed basement membrane and secretory cell disruptions (sections 7 and 8) that allowed SPZs a direct route (not requiring invasion) either to the lumen (step 7) or to the secretory cell cytoplasm (step 8). Some SPZs were seen in the secretory cell cytoplasm (sections 9 and 10), either as individuals (step 9) or groups (step 10). Rounded and/or fragmented SPZs (step 11) (36–38) were observed at the basement membrane and inside secretory cells. SPZs sometimes traversed secretory cell lateral cytoplasmic extensions en route to a neighboring secretory cavity (step 12). Other SPZs localized to pockets of lumenal saliva (step 13) were sometimes observed between the basement membrane and secretory cells (Fig. 1A, panel iv; see also Fig. 6B, panel ii). SPZs often bundled together when they reached a physical barrier, such as an aberrant WGA-positive chitinous wall (step 14) that sometimes lined the lumen (Fig. 5A). Some bundles cleared the barrier to enter the lumen (step 15), while in other cases, bundles represented a barrier that individual sporozoites navigated beyond (step 16). Some bundled SPZs traversed the basement membrane (step 17) (Fig. 6E, panel iv), exiting the SG and presumably entering the hemocoel. Certain SPZ groups, found primarily within secretory cavities, were captured by confocal imaging in a circling/swirling pattern (step 18) (Fig. 6B, panel vi; see also Movies S2 and S3).

In this report, we describe the many barriers to SG traversal by Plasmodium berghei SPZs presented by Anopheles stephensi SG architecture (Fig. 7). Typically, SPZs associated with the basement membrane (Fig. 7, step 1) invade SG secretory cells (Fig. 7, step 2), exit secretory cells and enter secretory cavities (Fig. 7, step 3), move from secretory cavities into the larger central lumen (Fig. 7, step 4), and enter the salivary duct through the open terminus (Fig. 7, step 5). We identified many variant architectural features with SGs that impeded sporozoite invasion, including fused salivary duct termini (Fig. 7, step 6), perilumenal chitin enrichments (Fig. 7, step 14, red asterisk), and bundled SPZs partially blocking the secretory cavity exit (Fig. 7, step 16). Accumulations of sporozoites were seen associated with these and other features, including secretory cell cytoplasms (Fig. 7, sections 9 and 10), aberrant basal compartments of saliva occurring between secretory cells and the basement membrane (Fig. 7, step 13), and circling arrangements within select secretory cavities (Fig. 7, step 18). Unnatural routes of entry or large secretory cell and/or basement membrane disruptions (Fig. 7, sections 7 and 8) might allow SPZs direct access to secretory cell cytoplasms, secretory cavities, or the lumen without secretory cell invasion in certain SGs. Abnormal sporozoite traversal events were seen in individuals across cell cytoplasms (Fig. 7, step 12) and as bundles moving through aberrant chitin accumulations (Fig. 7, step 15) or the basement membrane to exit the SG (Fig. 7, step 17). Rounded (possibly constrained/misshapen during invasion [36] or dead or dysregulated [37] or lysed [38]) SPZs (Fig. 7, step 11) were observed both in association with the basement membrane and inside some secretory cells. Thus, SG cell architecture plays a critical role in determining SPZ availability for transmission.

### Comparisons to prior studies.

Previous studies have used electron microscopy to analyze the localization of *Plasmodium* SPZs within infected SGs (12–15) as well as bundling (12). Posthuma and others observed Plasmodium falciparum SPZs invading (and inside) Anopheles stephensi SGs by electron microscopy (16, 39). Their results and ours largely agree. Their data suggest that SPZs pierce the basement membrane during invasion, as they showed no signs of membrane stretching or parasitophorous vacuole formation. Our data support this interpretation of the mechanism of entry. We did not observe membrane stretching. However, we did see that a halo of secretory cell cytoplasmic material was retained on the SPZ coat for some time after invasion (Fig. 3B, panel v, and C, panels iii and iv). Further, they observed CSP accumulation inside SG cells, such as we also observed (Fig. 3; see also Fig. S2 in the supplemental material). Thus, the different imaging techniques (immuno-EM and immunofluorescence) gave consistent results.

Pimenta and others provided the most complete description to date of the steps in SG invasion in Aedes aegypti infected with Plasmodium gallinaceum. Their model is still widely accepted and applied to many mosquito/*Plasmodium* combinations (12). In contrast to our data, they suggested that SPZs interact with the basement membrane and enter the SG through formation of a junction. During entry, the sporozoite acquires a vacuole of SG cellular material that is subsequently lost. Upon secretory cell exit, the sporozoite evaginates the apical membrane and enters the secretory cavity encased in a vacuole that is subsequently lost. Sporozoites then accumulate and bundle inside secretory cavities. Groups of bundled parasites then enter the salivary duct through an unknown mechanism that may involve a chitinase (12). Differences between our results and theirs likely reflect vector/parasite species differences.

Our results revealed both similarities and differences between *Aedes* SGs and *Anopheles* SGs with respect to *Plasmodium* invasion. Rather than a membranous vacuole forming around SPZs as they enter the SG cells using a moving junction (12), our findings and those reported previously others (16) suggest that SPZs puncture SG membranes and acquire a halo of cytoplasmic material that associates with the sticky SPZ coat following invasion (Fig. 3B, panel v, and C, panels iii to v) until it is lost by coat shedding. Both studies also determined that SPZs bundle inside secretory cavities, and we were able to capture both new small-scale SPZ interactions (Fig. 6A) and a new SPZ organization pattern, a circling/swirling pattern that could be a precursor to the bundles that were observed in both systems (Fig. 6B, panel vi; see also Movies S2 and S3 in the supplemental material). *Aedes* female SG duct termini are fused shut, making it unclear how sporozoites enter the salivary duct (12). We found fused duct termini in 3% of *Anopheles* SGs (24, 25) (this study) and very little evidence of how SPZs entered a closed duct. Whereas Pimenta and others suggested previously that hundreds of SPZs could occupy an *Aedes* SG secretory cell, we found that only about 40 SPZs could fill a typical *Anopheles* secretory cell cytoplasm (Fig. 1F). Further, we identified SPZ accumulations associated with many architecture features acting as barriers to SG traversal (Fig. 7).

### Connections across stages of the malaria cycle.

SPZ invasion was not always associated with large numbers of dying (cleaved caspase 3-positive) cells or basement membrane disruptions, even with large numbers (Fig. 5A). SPZs may reuse the same puncture sites for SG entry, without further SG damage, unless there are overwhelming numbers of sporozoites (Fig. 5B). Alternatively, SPZs may suppress apoptotic signals during initial invasion events, as happens during hepatocyte invasion (40). It may also be possible that the SGs can restore damaged basement membranes through deposition of new materials (12, 15, 16).

We identified sites in mosquito SG cells where the sporozoite motility and invasion protein CSP accumulates after it has been shed by the parasite during traversal. These sites (see Fig. S3 in the supplemental material), at the apical surface and a subcellular compartment relatively depleted of mitochondria and O-GlcNAcylated proteins, suggest possible interactions between CSP and the SG secretory cell. Singh and others found that shed CSP in hepatocytes blocked the production of NF-κB, dampening the immune response elicited by invaded cells (41). Further study will be required to determine if shed sporozoite CSP acts to dampen innate immunity in mosquito SGs. Interestingly, TRAP, like CSP, is also shed during motility and infection (35); however, we detected only one SPZ TRAP staining pattern (Fig. 6C).

10.1128/mBio.01238-19.3 FIG S3 Control staining demonstrating no SG staining with only secondary antisera. Scale bar length units are micrometers. Representative single-slice images from salivary glands (SGs) stained with only the secondary antibodies goat anti-chicken 488 (green) and either goat anti-rabbit or goat anti-mouse 647 (purple) dissected 23 days postinfection with P. berghei. SGs are visible by DIC light microscopy, and the microscope fluorescence channel settings used were consistent with those used for SG staining throughout the study. Download FIG S3, TIF file, 0.3 MB. Copyright © 2019 Wells and Andrew. 2019 Wells and Andrew This content is distributed under the terms of the Creative Commons Attribution 4.0 International license.

The extent to which SPZs communicate within mosquito SGs is unclear. Certain SPZ behaviors observed (bundling, bundle orientation, circling, traversing cellular barriers) suggest possible molecular communication. Other SPZ observations (consistent orientation toward the lumen, clustering at closed salivary duct termini) suggest that saliva flow and a possible molecular signal drive SPZs to leave the SGs.

### Membrane barriers and SPZ maturation in the SG.

Another key finding in our study is the existence of disruptions of large basement membranes, and sometimes secretory cells, in some infected SGs capable of allowing SPZs to bypass either or both of the required traversals of membranes (the basement membrane and the apical secretory cell membrane). Smaller disruptions had been observed previously (15, 16). This could facilitate mixed populations of SPZs within some SGs possessing mixed infection capability (thickly and thinly CSP-coated SPZs; Fig. 3A). SPZs may undergo a maturation process in the SGs to become capable of hepatocyte invasion in the mammalian host (42–44); SG membrane traversal may play a role in this process.

### Model for SG architecture as a roadblock to SPZ transmission.

The disparity between SG sporozoite numbers (reaching well into the thousands) and numbers of SPZs deposited into mammalian skin (10 to 100) has been well documented (22, 24). Work in other laboratories has suggested a high degree of transmission variability between individual mosquitoes (24). Further, pioneering live-imaging studies have also suggested that the first blood meal taken by mosquitoes capable of transmission is less successful than subsequent meals and that the SPZs that are transmitted already reside in the mouthparts just prior to probing (22). Our data suggest that SG architectural features limit the numbers of SPZs available for transmission.

Our current data suggest that SG invasion and traversal represent upstream, rate-limiting steps for parasite transmission. Once the route to the salivary duct is open, the pump muscle may force saliva (along with free SPZs) quickly through to the mouthparts, where SPZs then remain until the next probing event. Saliva flow is likely a major contributor to SPZ movement within SGs (22), but a diffusible chemical signal may also contribute (45). This signal could elicit the consistent orientation of SPZs toward the salivary duct that we observe in lumens bordered by aberrant chitin-positive regions (Fig. 1A, panel iv, and E, panel iii; see also Fig. 4A, panels iv to ix, and B, panels ii and iv).

## MATERIALS AND METHODS

### Mosquito husbandry.

Anopheles stephensi (Dutch strain) mosquitoes were maintained on 10% sucrose (available *ad libitum*) at 28°C and 75% humidity in a walk-in environmental chamber in the Johns Hopkins Bloomberg School of Public Health Malaria Research Institute Insectary using standard procedures (46). Sucrose was administered in a small glass bottle with a cotton wick prior to the first blood meal.

### Blood meals and infection.

Infective blood meals were conducted by JHMRI Insectary staff, primarily Godfree Mlambo, as previously described (47). After an infective blood meal, 10% sucrose was made continuously available to mosquitoes in a soaked cotton pad. In one experiment, a second, noninfective blood meal was similarly given to mosquitoes infected with Plasmodium berghei 23 days prior. The parasite strains used included the following: (i) wild-type (WT) P. berghei (ANKA strain; Fig. 1A, B, and E, Fig. 3, Fig. 4C and D, and Fig. 6A and E; see also Fig. S2 and Data Set S1 in the supplemental material) (48); (ii) P. berghei (ANKA strain) expressing GFP under the control of the elongation factor 1 alpha (eef1aa) promoter (Fig. 1; see also Fig. 2 and Fig. S1 [49]); (iii) P. berghei (ANKA strain) expressing GFP and luciferase under the control of the eef1aa promoter (Fig. 1F; see also Fig. 2A, G, and H, Fig. 4A and B, Fig. 5, and Fig. 6B and C and Fig. S3 [49]); and (iv) P. berghei (ANKA) expressing GFP under the control of the P. berghei HSP70 promoter (Fig. 1; see also Fig. 2 and Fig. S1 and Data Set S1 [50]). Similar results were observed across all strains tested.

10.1128/mBio.01238-19.4 DATA SET S1 Metadata and quantification (Microsoft Excel file). All metadata and counts correspond to staining and confocal microscopy of SG infection quantification. Raw image files are available upon request. Download Data Set S1, XLSX file, 1.7 MB. Copyright © 2019 Wells and Andrew. 2019 Wells and Andrew This content is distributed under the terms of the Creative Commons Attribution 4.0 International license.

### Dissections, staining, and mounting.

Salivary gland (SG) dissections, acetone permeabilization, tissue staining, and mounting were performed as described previously (27, 28). All primary antibodies were diluted 1:50, and all secondary antibodies were diluted 1:200. Secondary antibody staining in the absence of primary antibody staining showed very little background signal (see Fig. S3 in the supplemental material). Formaldehyde and glacial acetic acid fixation (28) was also tested (Data Set S1) to validate the results obtained by acetone permeabilization. Comparable results were observed. For the direct mounting experiment (Fig. 2; see also Fig. S2), dissected SGs, still attached to the head, were immediately added to a drop of glycerol on a microscope slide. Once 10 SGs were obtained, the tissues were positioned on the slide in glycerol and a cover slip was added.

### Dyes and antibodies.

The dyes and primary antisera employed in this study were as follows: DAPI (4′,6-diamidino-2-phenylindole; Life Technologies) (1:50), Rh-WGA (Vector Labs) (1:40), Nile red (Sigma) (1:50), phalloidin-Alexa 488 (Life Technologies) (1:10), chicken anti-GFP (Abcam ab13990), mouse anti-CSP (gift from Photini Sinnis), rabbit anti-mtTFA (Santa Cruz H-203), rabbit anti-TRAP (gift from Photini Sinnis), rabbit anti-CC3 (catalog no. 9661; Cell Signaling), AAPP (gift from Hiroyuki Matsuoka), and rabbit anti-GM130 (Abcam ab30637). Secondary antibodies (goat anti-rabbit 488 [A11008] and goat anti-mouse 647 [A21235]) were from Life Technologies.

### Confocal and bright-field microscopy.

Confocal imaging was conducted using a Zeiss LSM700 laser scanning confocal microscope housed in the Johns Hopkins University School of Medicine Microscope Facility. The step size used in three-dimensional (3D) image stack captures was 1 μm. Each figure is composed of representative images from between 6 and 20 imaged glands, selected from 30 to 120 dissected and stained glands per experiment, fixed, and immunostained between one and four diﬀerent days. DIC microscopy of the directly mounted samples was conducted on a Zeiss Axiophot light microscope equipped with ProgRes C14PLUS image capture system beginning within 2 h after the start of dissections.

### Image analysis and statistical analysis.

Image processing was completed in Zeiss Zen 2010, Adobe Photoshop CS4, and ImageJ (line scan). Quantification of sporozoite numbers and SG architecture features was done in Microsoft Excel. Statistical analyses and graphing were completed using Minitab 17 and Microsoft Excel. The original image stack capture files are available upon request. Raw data and analyses of infected SG quantification are provided in Data Set S1.
