# Supplementary figures and images for: Anopheles Salivary Gland Architecture Shapes Plasmodium Sporozoite Availability for Transmission
Source: mBio. 2019 Aug 6;10(4):e01238-19. doi: 10.1128/mBio.01238-19 (PMC6686039; doi:10.1128/mBio.01238-19)

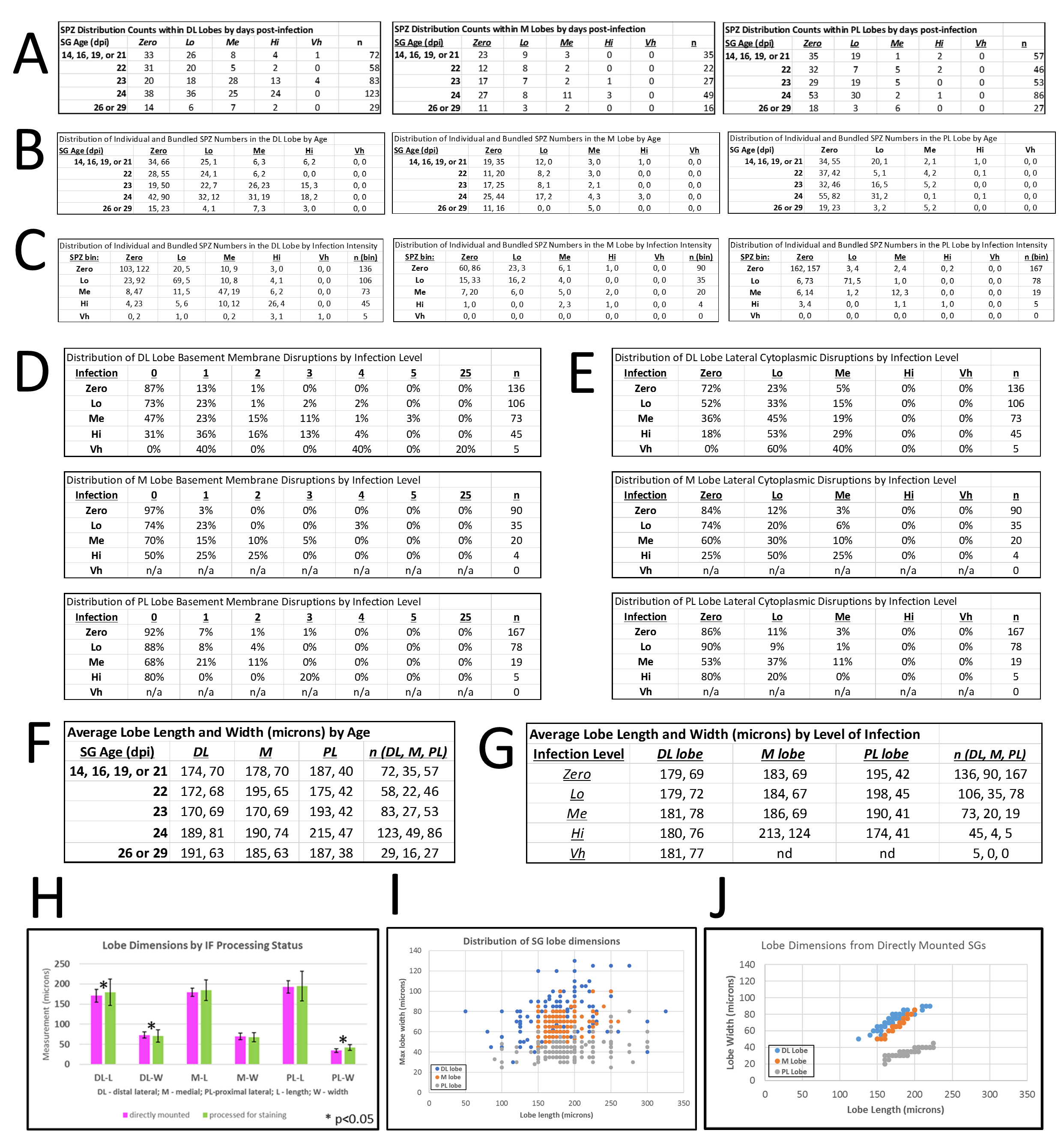

Supplement: FIG S1 [file mBio.01238-19-sf001.tif]

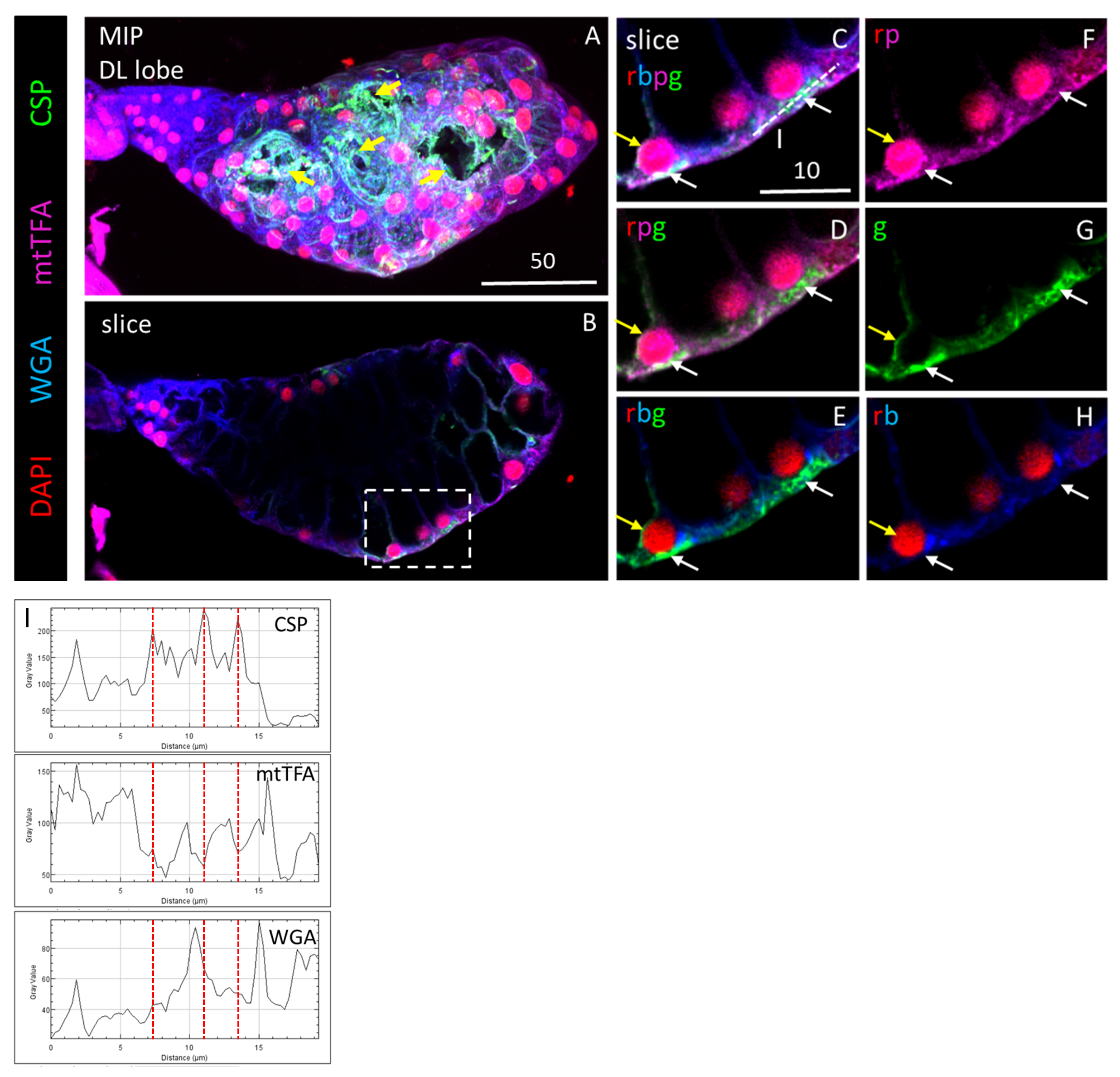

Supplement: FIG S2 [file mBio.01238-19-sf002.tif]

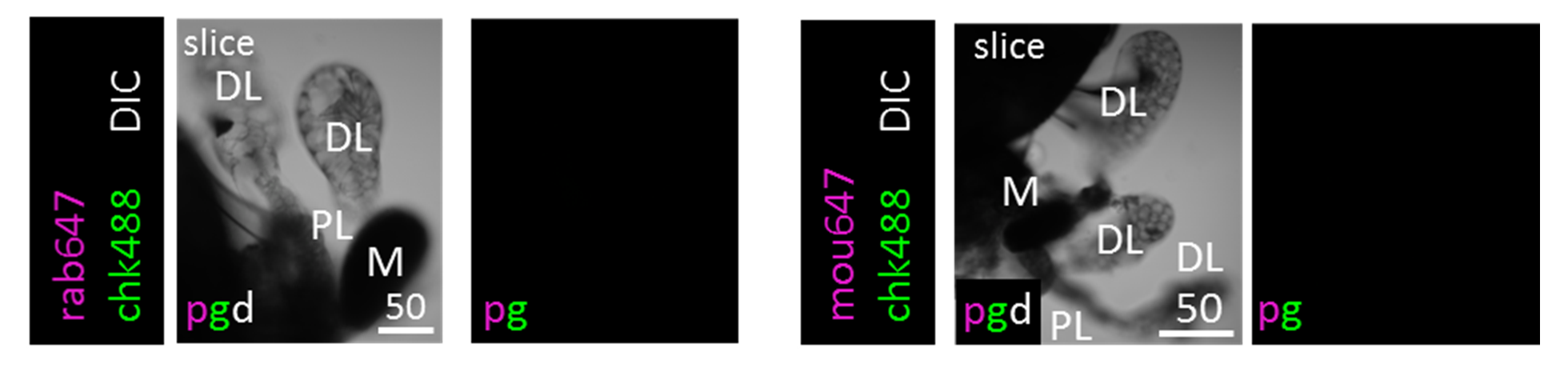

Supplement: FIG S3 [file mBio.01238-19-sf003.tif]
